# Whole exome sequencing of a family revealed a novel variant in the *CHM* gene, c.22delG p.(Glu8Serfs*4), which co-segregated with choroideremia

**DOI:** 10.1042/BSR20200067

**Published:** 2020-05-12

**Authors:** Handong Dan, Tuo Li, Xinlan Lei, Xin Huang, Yiqiao Xing, Yin Shen

**Affiliations:** 1 Eye Center, Renmin Hospital of Wuhan University, Wuhan 430060, Hubei, China; 2 Department of Ophthalmology, The Central Hospital of Enshi Tujia and Miao Autonomous Prefecture, Enshi Clinical College of Wuhan University, Enshi 445000, Hubei, China

**Keywords:** choroideremia, gene variant, whole exome sequencing

## Abstract

Choroideremia is a complex form of blindness-causing retinal degeneration. The aim of the present study was to investigate the pathogenic variant and molecular etiology associated with choroideremia in a Chinese family. All available family members underwent detailed ophthalmological examinations. Whole exome sequencing, bioinformatics analysis, Sanger sequencing, and co-segregation analysis of family members were used to validate sequencing data and confirm the presence of the disease-causing gene variant. The proband was diagnosed with choroideremia on the basis of clinical manifestations. Whole exome sequencing showed that the proband had a hemizygous variant in the *CHM* gene, c.22delG p. (Glu8Serfs*4), which was confirmed by Sanger sequencing and found to co-segregate with choroideremia. The variant was classified as likely pathogenic and has not previously been described. These results expand the spectrum of variants in the *CHM* gene, thus potentially enriching the understanding of the molecular basis of choroideremia. Moreover, they may provide insight for future choroideremia diagnosis and gene therapy.

## Introduction

Choroideremia (OMIM 303100) is a rare retinal degeneration that is characterized by the loss of retinal pigment epithelium (RPE), with subsequent loss of photoreceptors and choroid as the disease progresses [1]. The degeneration gradually progresses in a centripetal pattern with a pale fundus [2]. Although phenotypes may vary among patients, most typically complain of nyctalopia and constriction of the visual field at early stages, which eventually lead to complete blindness at late stages [3–5]. Although the structure and function of the central retina are altered at early stages, most patients retain intact visual acuity in the fifth decade of life [6,7]. The reported prevalence is approximately 1:50000 in the general population and inheritance mainly occurs in an X-linked recessive manner [8].

So far, variants affecting the *CHM* gene (OMIM 300390) are the sole known cause for choroideremia [9,10]. The *CHM* gene is located on chromosome Xq21.2 and contains 15 exons spanning 186382 bp; it encodes component A of the Rab geranylgeranyl transferase holoenzyme, Rab-escort protein 1 (REP-1). Rab is a GTP-binding protein that regulates post-translational isoprenyl modification and intracellular vesicular trafficking [11,12]. The Rab geranylgeranyl transferase holoenzyme activates the Rab GGTase subunit for catalysis of the geranylgeranyl transfer reaction. REP-1 is involved in prenylation of Rabs in rods and RPE [13–15] and binds unprenylated Rab GTPases. Rab GTPases need to be geranylgeranylated on either one or two cysteine residues in their C-terminus to localize to the correct intracellular membrane. REP-1 is a key mediator of membrane trafficking and an essential component of the catalytic Rab geranylgeranyl transferase II complex in retina. Any disease-causing variant of the *CHM* gene can lead to a loss of geranylgeranyl transferase function and insufficient transfer of geranylgeranyl pyrophosphate groups on to Rab proteins. Variants in the *CHM* gene can lead to a deficiency of REP-1 and the onset of chorioretinal atrophy [16].

In the present study, we analyzed three generations of a Chinese family; we performed routine ophthalmological examinations and whole exome sequencing to investigate the pathogenic variant and molecular etiology associated with choroideremia in the family. The proband was diagnosed with choroideremia and had a novel hemizygous variant, c.22delG p. (Glu8Serfs*4), in the *CHM* gene.

## Materials and methods

### Subjects

All participants were recruited at Renmin Hospital of Wuhan University. The present study was authorized by the Institutional Review Board of Renmin Hospital of Wuhan University. Prior to the study, written informed consent was obtained from each participant or legal guardian, in accordance with the tenets of the Declaration of Helsinki.

### Clinical examination

Exhaustive family histories, clinical data, and peripheral blood samples were obtained from all available family members. Routine ophthalmological examinations were performed, including assessment of best-corrected visual acuity, refractive error, intraocular pressure, slit-lamp microscopy, and ophthalmoscopy. Selected family members underwent additional ophthalmic examinations including high-resolution fundus photography, spectral-domain optical coherence tomography (SD-OCT) and full-field electroretinography (ERG). Fundus photographs were obtained with a digital fundus camera VISUCAM 200 (Carl Zeiss Meditec AG, Jena, Thuringia, Germany). SD-OCT was performed using an AngioVue® Imaging System (Optovue, Fremont, California, United States). Full-field ERG was recorded using an Espion System (Diagnosys, Westford, Massachusetts, United States), in accordance with the standards and methodology of the International Society for Clinical Electrophysiology of Vision [17].

### Whole exome sequencing

Genomic DNA of family members was isolated from leukocytes in venous blood samples by using the TIANamp Blood DNA Midi Kit (TIANGEN Biotech, Beijing, China), in accordance with the manufacturer’s instructions. Paired-end multiplex libraries of the proband were constructed using xGen Exome Research Panel v1.0 (Integrated DNA Technologies, Coralville, Iowa, United States), in accordance with the manufacturer’s instructions. Sequencing enrichment was performed on a HiSeq platform (Illumina, San Diego, California, United States) to generate 150–200-bp paired-end reads, in accordance with the manufacturer’s protocol [18,19]. Genomic DNA data were analyzed via whole exome sequencing. Sequence alignments were performed using the Burrows–Wheeler Aligner (http://bio–bwa.sourceforge.net/) [20]. Variant calling and annotation were conducted in accordance with previously reported protocols [21].

### *In silico* analyses

Analysis-ready alignment data were acquired by filtering raw reads to remove duplicates and performing local alignment to the hg19 (GRCh37) human reference sequence. Base quality was recalibrated by Picard Mark Duplicates (http://sourceforge.net/projects/picard/), Genome Analysis Toolkit (https://gatk.broadinstitute.org/hc/en-us), and SAM tools (http://samtools.sourceforge.net/) [22]. Single nucleotide variations, insertions, and deletions were identified using the Genome Analysis Toolkit. Copy number variants were detected using the Weaver algorithm by comparing average depth between the proband and normal human samples [23]. An allele frequency chart was used to calculate the allele frequency of each locus in the target region. Variants were preferentially selected for further analysis and validation if they met all of the following criteria: (i) minor allele frequency < 0.01 in the 1000 Genomes Project database (http://www.internationalgenome.org/), Exome Aggregation Consortium database (ExAC, http://exac.broadinstitute.org/), and Genome Aggregation database (gnomAD, http://gnomad.broadinstitute.org/); (ii) occurrence in exon regions or canonical splicing sites that affected RNA splicing; (iii) potential functional effects of nonsynonymous single nucleotide variants were predicted to be damaging or deleterious using multiple lines of computational prediction; (iv) candidate gene variants related to ophthalmic hereditary disease, especially for inherited retinal disease; (v) other reported potential pathogenic variants that did not met the above criteria (e.g., high minor allele frequency variants, deep-intronic variants, and synonymous single nucleotide variants). Variant nomenclature complied with the recommendations of the Human Genome Variation Society (HGVS, http://www.hgvs.org/) [24]. Variant annotation complied with the guidelines of the American College of Medical Genetics (ACMG, https://www.acmg.net/) [25,26].

### Sanger sequencing and co-segregation analysis

The preferentially selected variants were validated and co-segregation was analyzed by Sanger sequencing. The polymerase chain reaction was used to amplify *CHM* gene fragments that included variants with the forward primer CCCAAAACTCGCCACTGACAGA and reverse primer CACAGAGCAAACCGCCTTCAATT using 3500xL Dx Genetic Analyser (Applied Biosystems, Foster City, California, United States) with the ABI BigDye Terminator v3.1 Cycle Sequencing kit (Applied Biosystems, Foster City, California, United States). Primers were designed with Primer 3 (http://primer3.ut.ee/). Participants’ sequences and corresponding consensus sequences (obtained from the NCBI Human Genome Database: https://www.ncbi.nlm.nih.gov/) were analyzed using SeqMan II software in the Lasergene software package (DNASTAR, Madison, Wisconsin, United States).

## Results

### Clinical manifestations

**Figure 1:**
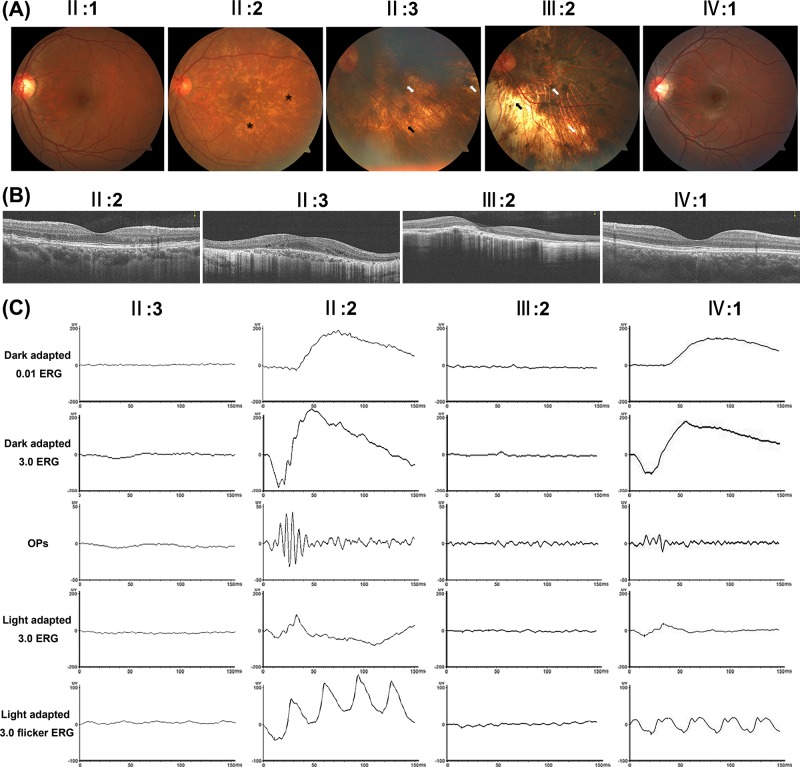
Typical fundus, SD-OCT, and ERG findings of the family members (**A**) Typical fundus of proband’s uncle (II:3) and proband (III:2) exhibited fundus signs indicative of choroideremia including confluent RPE atrophy, choriocapillaris loss, widespread depigmentation around mid-peripheral or centripetal retina, and choroidal vessels in exposed choroid areas. Fundus of proband’s mother (II:2) showed numerous scattered pigmentary changes, patchy areas of chorioretinal degeneration, and crystal-like retina appearance. Fundus of proband’s father (II:1) and daughter (IV:1) revealed normal findings. Asterisks indicate scattered pigmentary changes, chorioretinal degeneration, and crystal-like retina appearance; single black arrowheads indicate RPE atrophy and choriocapillaris loss; single white arrowheads indicate depigmentation around retina; double white arrowheads indicate choroidal vessels in exposed choroid areas. (**B**) SD-OCT of proband’s uncle (II:3) and proband (III:2) revealed thinning of the retina and choroid at the macular fovea, increased signal transmission posterior to RPE and Bruch’s membrane, and absence of photoreceptor-attributable reflectivity bands. SD-OCT of proband’s mother (II:2) and daughter (IV:1) revealed normal findings. (**C**) Full-field ERG of proband’s uncle (II:3) and proband (III:2) demonstrated extinguished dark-adapted and light-adapted responses. Full-field ERG of proband’s mother (II:2) and daughter (IV:1) revealed normal findings.

In total, seven family members were enrolled in the study. Patients II:3 and III:2 complained of night blindness, constricted visual field, and impaired vision in the first decade of life. They exhibited fundus signs typical of choroideremia including confluent RPE atrophy, choriocapillaris loss, widespread depigmentation around mid-peripheral or centripetal retina, and choroidal vessels in exposed choroid areas. SD-OCT revealed thinning of the retina and choroid at the macular fovea, increased signal transmission posterior to RPE and Bruch’s membrane, and absence of photoreceptor-attributable reflectivity bands. Full-field ERG demonstrated extinguished dark-adapted and light-adapted responses. The proband’s mother (II:2) complained of mild nyctalopia with normal visual acuity; fundus examination showed numerous scattered pigmentary changes, patchy areas of chorioretinal degeneration, and crystal-like retina appearance. SD-OCT and full-field ERG findings were normal. The proband’s daughter (IV:1) did not complain of any symptoms; her fundus, SD-OCT, and full-field ERG examinations revealed normal findings. The proband’s father (II:1) did not complain of any symptoms; his fundus examination revealed normal findings. Typical fundus, SD-OCT, and ERG findings of family members are shown in Figure 1. Clinical features and *CHM* variant information among the family members are summarized in Table 1. Based on the clinical manifestations, the proband was diagnosed with choroideremia.

**Table 1:** Clinical features and *CHM* variant information among the family members

ID	Gender	Symptom	Age at (year)		BCVA		Fundus examination	SD-OCT	ERG	Nucleotide change	Amino acid change	Het/ Hem
			Onset	Exam	OD	OS						
II:1	Male	No	-	56	1.0	1.0	Normal	NA	NA	WT	-	-
II:2	Female	Nyctalopia	-	55	1.0	1.0	Pigmentary changes, chorioretinal degeneration, crystal-like retina appearance	Normal	Normal	c.22delG	p. (Glu8Serfs*4)	Het
II:3	Male	Nyctalopia, vision decline, vision field defect	7	53	HM	HM	RPE atrophy, choriocapillaris loss, depigmentation	Thinning	Extinguished	c.22delG	p. (Glu8Serfs*4)	Hem
II:5	Female	No	-	48	1.0	1.0	Normal	NA	NA	WT	-	-
III:2	Male	Nyctalopia, vision decline, vision field defect	5	30	HM	HM	RPE atrophy, choriocapillaris loss, depigmentation	Thinning	Extinguished	c.22delG	p. (Glu8Serfs*4)	Hem
III:3	Female	No	-	25	1.0	1.0	Normal	NA	NA	WT	-	-
IV:1	Female	No	-	7	1.0	1.0	Normal	Normal	Normal	c.22delG	p. (Glu8Serfs*4)	Het

Abbreviations: BCVA, best-corrected visual acuity; Hem, hemizygous; Het, heterozygous; HM, hand movement; NA, not available; OD, right eye; OS, left eye; WT, wild-type.

### Whole exome sequencing results

**Figure 2:**
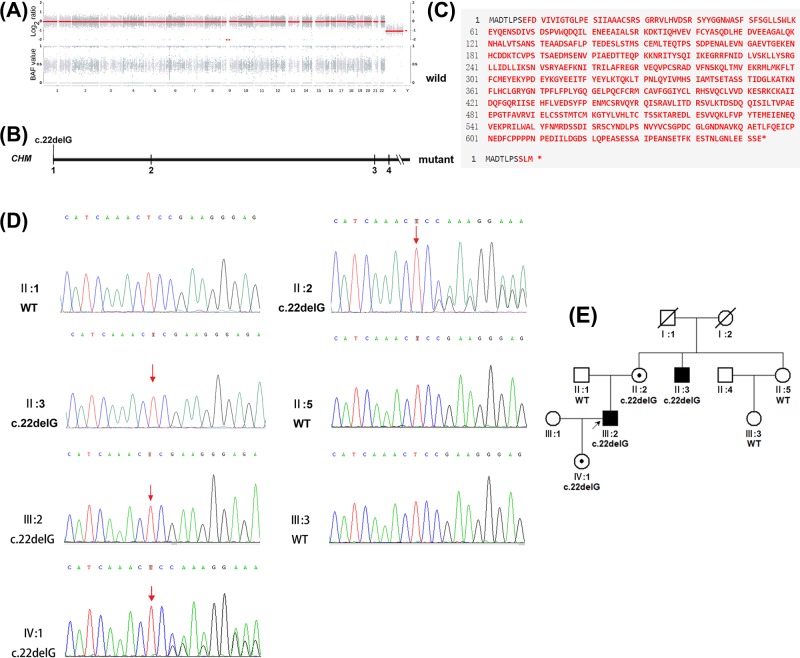
Whole exome sequencing results of the family (**A**) Copy number and allele frequency charts show that no copy number variants associated with the phenotype were found in the proband. Upper part shows log2 values of copy numbers of 23 pairs of chromosomes, and lower part shows allele frequency distributions of these chromosomes. B-allele frequency value of 1 represents homozygous variant, 0.5 represents heterozygous variant, and 0 represents a genotype identical to the reference genome. Numbers and letters below diagram denote chromosomes. (**B**) Schematic representation of the genomic structure of the *CHM* gene showing the location of novel variant c.22delG p. (Glu8Serfs*4). Numbers below diagram indicate corresponding exon numbers. Parts of exons are omitted. (**C**) The effect of the variant on the generation of a predicted premature termination codon. The variant replaced glutamic acid with serine at the eighth amino acid, which may lead to generate a premature termination codon at residue 12. Upper part shows wild-type amino acid sequence, lower part shows mutant amino acid sequence. (**D**) Sanger sequencing chromatographs of seven family members showed that the proband (III:2), his mother (II:2), uncle (II:3), and daughter (IV:1) carried the hemizygous c.22delG variant in the *CHM* gene; the proband’s father (II:1), aunt (II:5), and cousin (III:3) did not carry the c.22delG variant in the *CHM* gene. Arrows denote mutant base. Notably, the sequencing chromatographs showed the reverse complementary sequences of c.22delG. (**E**) Pedigree of the family with c.22delG variant. The pedigree showed that the variant was completely co-segregated with the choroideremia in this family. Circles denotes unaffected females, boxes denote unaffected males, symbols with slashes indicate deceased family members, symbols with dark spots indicate carriers, dark symbols indicate affected participants, and arrow denotes proband.

Average sequencing depth and coverages of the whole exome were computed on all target regions designed to cover 19396 genes of human genome according to product information provided by the manufacturer. It showed that the average depth is 103, with 99.03 and 98.99% of the entire target region covered over 20× and 30×, respectively. In total, 45.7 million reads mapped to the human reference genome; 85738 variants were called in the proband sample, consisting of 71724 single nucleotide variants and 14014 insertions and deletions. No copy number variants associated with the phenotype were found in the proband. The copy number and allele frequency charts are shown in Figure 2A.

Based on a comparative analysis of whole exome sequencing data and the use of a prioritization program, we determined that the proband (III:2) had four variants that were likely pathogenic or of uncertain significance. These variants were as follows: c.22delG p.(Glu8Serfs*4) in the *CHM* gene, c.8502A>T p.(Glu2834Asp) in the *USH2A* gene, c.950T>C p.(Ile317Thr) in the *NHLRC2* gene, and c.2975-6G>A in the *CACNA2D4* gene. Potential functional effects of c.8502A>T in the *USH2A* gene and c.950T>C in the *NHLRC2* gene were predicted to be tolerated or benign using multiple lines of computational prediction. The variants of c.22delG in the *CHM* gene and c.2975-6G>A in the *CACNA2D4* gene are not frequently found in an ethnically matched population from East Asia, whereas the variants of c.950T>C in the *NHLRC2* gene and c.2975-6G>A in the *CACNA2D4* gene are frequently found in that population. Predictive functional effects and population distribution frequencies of these variants are summarized in Table 2. So, the hemizygous variant of c.22delG p. (Glu8Serfs*4) in the *CHM* gene is the most preferentially selected variant for further analysis. The variant is located in the first exon of the *CHM* gene. A schematic representation of the genomic structure of the *CHM* gene is shown in Figure 2B. The variant replaced glutamic acid with serine at the eighth amino acid, which may lead to generate a premature termination codon at residue 12. The effect of the variant on the generation of a predicted premature termination codon is shown in Figure 2C. Co-segregation analysis by Sanger sequencing showed that the proband's mother (II:2), uncle (II:3), and daughter (IV:1) carried the hemizygous variant, c.22delG p. (Glu8Serfs*4); in contrast, the proband's father (II:1), aunt (II:5), and cousin (III:3) did not carry the c.22delG p. (Glu8Serfs*4) variant. The Sanger sequencing chromatographs of seven family members are shown in Figure 2D. The variant was completely co-segregated with the choroideremia in this family. The pedigree of the family is shown in Figure 2E. In addition, the variant was a null variant that was predicted to disrupt gene function by causing complete absence of the gene product. Based on the above findings, we concluded that the variant c.22delG p. (Glu8Serfs*4) was likely to be pathogenic. The variant may lead to formation of a premature termination codon and presumably nonsense-mediated mRNA decay; this is predicted to produce a truncated REP-1 protein that exhibits loss of function. Thus, the variant c.22delG p. (Glu8Serfs*4) in the *CHM* gene is the genetic etiological factor for this family.

**Table 2:** Predictive functional effects and population distribution frequencies of four variants that were likely pathogenic or of uncertain significance

Gene	Location	Transcript	Variation	SIFT	PolyPhen2 HDIV	PolyPhen2 HVar	ClinPred	LRT	Mutation Taster	Mutation Assessor	FATHMM	PROVEAN	GERP	1000 Genomes	ExAC	GnomAD (exome)	gnomAD (genome)
*CHM*	chrX:85302514	NM_000390.2	c.22del p.(Glu8SerfsTer4)	NA	NA	NA	NA	NA	NA	NA	NA	NA	NA	NA	NA	NA	NA
*USH2A*	chr1:216052162	NM_206933.2	c.8502A>T p.(Glu2834Asp)	0.036	0.235	0.146	0.32874178	0.009404	0.9779	2.01	0.38	-1.55	-0.874	NA	NA	NA	NA
*NHLRC2*	chr10:115644050	NM_198514.3	c.950T>C (p.Ile317Thr)	1	0.001	0.004	0.04571371	0.009133	0.6465	-1.82	1.27	3.07	5.03	0.002	0.0007	0.00093	0.0019
*CACNA2D4*	chr12:1908867	NM_172364.4	c.2975-6G>A	NA	NA	NA	NA	NA	NA	NA	NA	NA	NA	0.001	0.0008	0.000078	0.0006

SIFT, deleterious (≤0.05), tolerated (>0.05); PolyPhen2HDIV: probably damaging (≥0.957), possibly damaging (0.453−0.956), benign (≤0.452); PolyPhen2Hvar: probably damaging (≥0.909), possibly damaging (0.447–0.909), benign (≤0.446); ClinPred: deleterious (≥0.5), tolerated (<0.5); LRT: lower scores are more deleterious; Mutation Taster: higher values are more deleterious; Mutation Assessor: higher values are more deleterious; FATHMM: lower values are more deleterious; PROVEAN: higher values are more deleterious; GERP: it ranges from −12.3 to 6.17, with 6.17 being the most conserved. Abbreviation: NA, not available.

## Discussion

Patients with choroideremia typically present with early nyctalopia, but the majority retain excellent visual acuity until the end stages of disease. This preservation of visual acuity is potentially the result of continuing Müller cell function, which contributes to the cone visual cycle in the absence of RPE [27,28]. Female carriers of choroideremia typically exhibit a clinically heterogeneous phenotype and display variable severity of the disease due to random X-chromosome inactivation. In this state, cells carrying variant alleles are intermixed with cells expressing normal X chromosomes, thereby leading to a mosaic pattern of the disease [29]. Although both the proband’s mother and daughter carried the hemizygous variant, c.22delG, the proband’s mother exhibited a mild abnormal fundus appearance, whereas the proband’s daughter exhibited a normal fundus appearance. This discrepancy is presumably because retinopathy was below the range of detection in the proband’s young daughter; in addition, some female carriers may exhibit normal clinical manifestations throughout life.

In the study, we detected a novel hemizygous variant, c.22delG p. (Glu8Serfs*4) in the *CHM* gene. The frameshift variant was predicted to create a premature stop codon and lack of full-length REP-1. This would presumably cause a deficiency of geranylgeranyl transferase function and insufficient transfer of geranylgeranyl pyrophosphate groups on to Rab proteins, leading to abnormal protein function and prevention of their participation in intracellular vesicular transport. The ultimate outcomes of these changes would include several clinical manifestations, as reported in the study.

Thus far, 309 disease-associated variants in the *CHM* gene have been identified in patients with choroideremia, including 84 missense/nonsense variants, 51 splicing variants, 71 small deletion variants, 26 small insertion variants, 10 small indel variants, 55 gross deletion variants, 3 gross insertion variants, 4 regulatory variants, and 5 complex rearrangement variants (https://www.hgmd.cf.ac.uk, updated 2019.3). The majority of the reported variants are null variants that can facilitate gene replacement therapy for patients [30,31]. Most nonsynonymous variants involved C to T transition, which produces a higher number of CpG dinucleotides [32]. This transition can result in a premature stop codon due to arginine residue substitution, thereby considerably truncating the REP-1 protein. This transition is a well-known trigger of variants in the human genome and has occurred with high frequency throughout evolution because of inherent instability via methylation [33]. Exons 5, 6, and 11 are the most commonly affected exons [34]; in this study, the variant was located in exon 1.

Traditionally, choroideremia is typically diagnosed on the basis of family history and the results of clinical examination. Most patients with choroideremia have a characteristic fundus phenotype, which includes RPE atrophy, choriocapillaris loss, retina depigmentation, and choroidal vessels in exposed choroid areas [35]; this phenotype differs from the phenotype observed in patients with retinitis pigmentosa, which includes bone spicule pigmentation, retinal vascular stenosis, and a waxy-pale optic disc [36]. Both patients with retinitis pigmentosa and patients with choroideremia often complain of nyctalopia, visual field restriction, and reduced visual acuity, including blindness in the late stages of disease [37,38]; in addition, some female carriers show mild phenotypes due to a mosaic X-linked inheritance pattern. Therefore, it is difficult to achieve an accurate diagnosis for some patients with atypical choroideremia [39] and choroideremia is often misdiagnosed as retinitis pigmentosa [40]. Exome sequencing is a powerful tool to identify causative variants and distinguish between choroideremia and retinitis pigmentosa [41,42]. In a study by Li et al., exome sequencing helped to achieve a revised diagnosis for six probands with atypical choroideremia, all of whom were initially misdiagnosed with retinitis pigmentosa [43]. Genetic sequencing can clarify the diagnosis and identify up to 94% of disease-causing variants in patients with choroideremia [44]. Whole exome sequencing is a non-invasive and non-biased diagnostic method that can accurately diagnose inherited eye diseases with complex clinical manifestations [45,46]. This approach is more efficient and cost-effective than Sanger sequencing and whole-genome sequencing [47–49]. Therefore, whole exome sequencing is extremely useful for the identification of genetic diseases by clinicians and researchers.

It was reasonable to conclude that the likely pathogenic variant c.22delG p. (Glu8Serfs*4) was the disease-causing variant of the family on the basis of co-segregation analysis, multiple lines of computational prediction, and absence of the variant in a control population; however, there was a notable limitation in the present study. We enrolled a small family, rather than a large, multigenerational family. Because of the complex inheritance, atypical clinical presentation of patients, and mild phenotypes of female carriers, a small pedigree is not sufficient to disentangle the genetic etiology of choroideremia. Thus, we plan to enroll multiple families in future research studies.

In conclusion, we analyzed a Chinese family with night blindness, constricted vision field, and impaired vision. The proband was diagnosed with choroideremia based on the findings of ophthalmological examinations. Through whole exome sequencing, we found that the proband had a hemizygous variant in the *CHM* gene, c.22delG p. (Glu8Serfs*4), which has not previously been reported. Sanger sequencing showed that the variant co-segregated with choroideremia within the family. These results expand the spectrum of variants in the *CHM* gene, thus enriching the understanding of the molecular basis, family genetic counseling, and clinical management of choroideremia.

## Data Availability

They are available on special request.

## Abbreviations

ERG: electroretinography
REP-1: Rab-escort protein 1
RPE: retinal pigment epithelium
SD-OCT: spectral-domain optical coherence tomography
